# Biology and Application of *Chaetomium globosum* as a Biocontrol Agent: Current Status and Future Prospects

**DOI:** 10.3390/microorganisms13071646

**Published:** 2025-07-11

**Authors:** Shailja Sharma, Saurabh Pandey, Sourabh Kulshreshtha, Mukesh Dubey

**Affiliations:** 1 Faculty of Forensic Sciences, Mandsaur University, SH-31, Mhow–Neemuch By-Pass Square, Rewas-Dewda Road, Mandsaur 458001, India; 2 Ministry of Ayush, Ayush Bhawan, New Delhi 110023, India; 3 Faculty of Applied Sciences and Biotechnology, Shoolini University of Biotechnology and Management Sciences, Solan 173229, India; 4 Department of Forest Mycology and Plant Pathology, Swedish University of Agricultural Sciences, 75007 Uppsala, Sweden

**Keywords:** antibiosis, mycoparasitism, induced defense response, interference competition, microbiome, plant health promotion

## Abstract

*Chaetomium globosum* is a widely distributed fungal species recognized for its ability to produce a range of secondary metabolites. This fungus plays a significant ecological role by degrading organic matter and contributing to nutrient cycling in diverse ecosystems. In recent years, *C. globosum* has attracted considerable scientific interest due to its potential as a biocontrol agent [BCA] against a wide array of diseases in numerous plant species. While the precise mechanisms of *C. globosum* as a BCA remain poorly understood, interference competition through antibiosis is one of the key mechanisms. Moreover, *C. globosum* can enhance plant health by promoting nutrient availability, manipulating the rhizosphere microbiome, and inducing plant defense responses. The formulation of *C. globosum* for agricultural applications has been reported, which can significantly improve stability and efficacy under field conditions. However, despite significant advancements in omics and molecular biology technologies, the biology of *C. globosum* is understudied. Enhanced research into the genetics and functional genomics of *C. globosum* could pave the way for its applications in sustainable agriculture. This review summarizes the role of *C. globosum* as a BCA, focusing on its underlying mechanisms such as genomics and transcriptomics, and the effects of *C. globosum* application on soil health and the rhizosphere microbiome.

## 1. Introduction

*Chaetomium globosum* is a saprotrophic filamentous fungus which belongs to the family Chaetomiaceae under the order Sordariales. Known for its strong cellulolytic activity, this fungus is widespread across various environments and ecological niches, mainly thriving in soil, decomposing organic matter, and cellulose-rich plant material. It plays a crucial role in nutrient cycling in ecosystems. Beyond its ecological significance, *C. globosum* has attracted substantial interest due to its production of a range of compounds with activity against various pathogens, including fungi and nematodes [1,2,3]. In addition, *C. globosum* can parasitize fungal hosts and colonize plant roots, promoting plant growth and defense responses [4,5]. Due to these attributes, this fungus is recognized as a promising biological control agent [BCA] against root and foliar fungal plant pathogens and nematodes responsible for various plant diseases affecting crop yields and quality [4,5]. This review provides an in-depth examination of several critical areas of *C. globosum,* including its mode of action as a BCA and its underlying mechanisms, including genomics and transcriptomics. Additionally, this review explores the advancement in *C. globosum* taxonomy, and formulation for large-scale field and greenhouse applications.

## 2. *Chaetomium globosum* as an Effective Biocontrol Agent

The term “biological control” [or “biocontrol”] refers to the management of various types of pests, including insect pests, crop pathogens, weeds, and nematodes, using naturally occurring microbes [6]. Over the past century, the practices and concepts of biological control have evolved into distinct streams, each associated with specific scientific and taxonomic disciplines. Parallel developments have increased references to biological control in industrial contexts and legislation, resulting in conceptual and terminological fragmentation. Aligning with recent terminology updates proposed by Sternberg et al. [6], this review defines biological control as the use of living organisms to manage plant pathogens and diseases. While BCAs refer specifically to living organisms, products based on non-living, nature-derived substances are considered a separate category under the umbrella of bio-protection [6]. Based on its application, biological control can be classified into four categories: natural biological control, conservation biological control, classical biological control, and augmentative biological control. Natural and conservation biocontrol involve resident organisms with or without human interventions, respectively. Classical and augmentative biocontrol consists of the application of additional organisms with the intention of their permanent and temporary establishment, respectively [6].

*Chaetomium globosum* has been identified as an effective BCA against a variety of soil- and air-borne fungal and oomycete plant pathogens that cause foliar and root diseases, both in greenhouse and field conditions [4,5]. Notable examples of these diseases include wheat and barley spot blotch caused by *Bipolaris sorokiniana* [7,8]; ascochyta blight disease of chickpea caused by *Ascochyta rabiei* [9]; root rot in citrus caused by *Phytophthora nicotianae* [10]; tomato leaf spot disease caused by *Alternaria alternata* [11]; potato late blight disease caused by *Phytophthora infestans* [12]; root rot of date palm caused by *Rhizoctonia solani*, *Fusarium oxysporum*, *Fusarium chlamydosporum*, and *Neocosmospora solani* [13]; and fusarium crown rot [FCR] symptoms in wheat seedlings [14].

*Chaetomium globosum* has been used as a seed treatment to combat soil-borne diseases affecting corn seedlings caused by *Fusarium roseum* [15,16]. Similarly, wheat seeds coated with spore suspensions have exhibited improved germination rates, reduced fusarium root rot disease, and increased wheat yields [14]. In growth chamber experiments, an ascospore suspension of *C. globosum* effectively controlled apple scab caused by *Venturia inaequalis* (Cke.) Wint. when applied as a foliar treatment [17,18]. Similarly, a *C. globosum* formulation composed of colloidal cellulose showed a significant biocontrol effect against apple scab, flyspeck (caused by *Zygophiala jamaicensis E. Mason*), and sooty blotch (caused by *Phyllachora pomigena*) under greenhouse and field conditions [19]. Biocontrol of *Alternaria raphani* and *Alternaria brassicicola* on radish seedlings and pod infection and Apple scab disease caused by *V. inaequalis* by *C. globosum* has also been reported [20]. In addition, spray application of *C. globosum* ascospore suspension in the open field significantly reduces the perithecial formation and survival of the soybean stem canker pathogen *Diaporthe phaseolorum f. sp. meridionalis* on soybean stubble [21]. A summary of plant diseases controlled by *C. globosum* under greenhouse and field conditions is given in Table 1.

The biocontrol effect of *C. globosum* against various plant-parasitic nematodes is also shown. For example, tuber treatment and soil application of *C. globosum* reduced potato cyst nematode *Globodera rostochiensis* [42]. Similarly, seed treatment of *C. globosum* reduced gall formation in cucumber seedlings [43], inhibited the root knot nematode (RKN) *Meloidogyne incognita* infection, and reduced female reproduction in cotton roots [44,45]. 

The following report presents case studies that illustrate the successful use of *C. globosum* in combating root and crown rot, as well as foliar pathogens. These examples highlight its effectiveness under both field and polyhouse conditions.

Field trials involving *C. globosum,* two wheat varieties (Aikang 58 and Bainong 207), and the FCR pathogen *Fusarium pseudograminearum* were conducted at three locations (Kaifeng, Wenxian, and Neihuang) in China between 2018 and 2022. The results exhibited variable biocontrol efficacy [26–73%] depending on the year, location, and wheat variety used [14]. During the 2018 field trials at Kaifeng, wheat seeds from the variety Bainong 207 treated with *C. globosum* showed a significant 43% reduction in FCR disease and a 3.2% increase in yield compared to the control. During the field trials in China using two wheat varieties Aikang 58 and Bainong 207 at two locations [Wenxian and Neihuang], *C. globosum* treatments resulted in a significant reduction in FCR disease by 70% and 28.0%. This resulted in an increase of 10.7% and 11.9% in yield, respectively, compared to the control [14]. These results are comparable to those obtained using a seed coating treatment with the chemical agent difenoconazole, which was used as a positive control in the same experiment.

Two field trials were conducted in the regions of Perar and Nanjanad, Nilgiris, Tamil Nadu, India, to evaluate the effectiveness of the *C. globosum* liquid formulation against potato late blight disease caused by the oomycete pathogen *P. infestans* [12]. The liquid formulation was applied as a tuber treatment, soil application, and two foliar spray applications, individually or in combination. The results showed an average reduction of 20–40% in the late blight disease index compared to the untreated control, which exhibited a 100% disease index. The combined application of tuber, soil, and foliar treatments demonstrated a better biocontrol effect than individual applications. Furthermore, the application of *C. globosum* enhanced tuber yield (averaging 28.3 t/ha) compared to the untreated control (16.0 t/ha). The biocontrol efficacy of *C. globosum* was compared to fungicide application, where metalaxyl + mancozeb (0.2%) was used as a tuber treatment and applied twice as foliar treatments. However, tuber yield was comparatively higher with the chemical treatment (31.3 t/ha) [12].

Varsha and co-authors evaluated the biocontrol efficacy of the liquid formulation of *C. globosum* alone and in combination with an arbuscular mycorrhizal fungus (AMF) against the soil-borne pathogen *Macrophomina phaseolina* on strawberries under polyhouse conditions in Ooty, Tamil Nadu, India [46]. The application of *C. globosum* formulation via basal application, seedling dip, and soil drenching decreased *M. phaseolina* root rot disease by 51% compared to the control. However, the combined application of *C. globosum* and AMF resulted in a 74% higher disease reduction [46].

## 3. Biocontrol Mechanism of *Chaetomium globosum*

**Figure 1:**
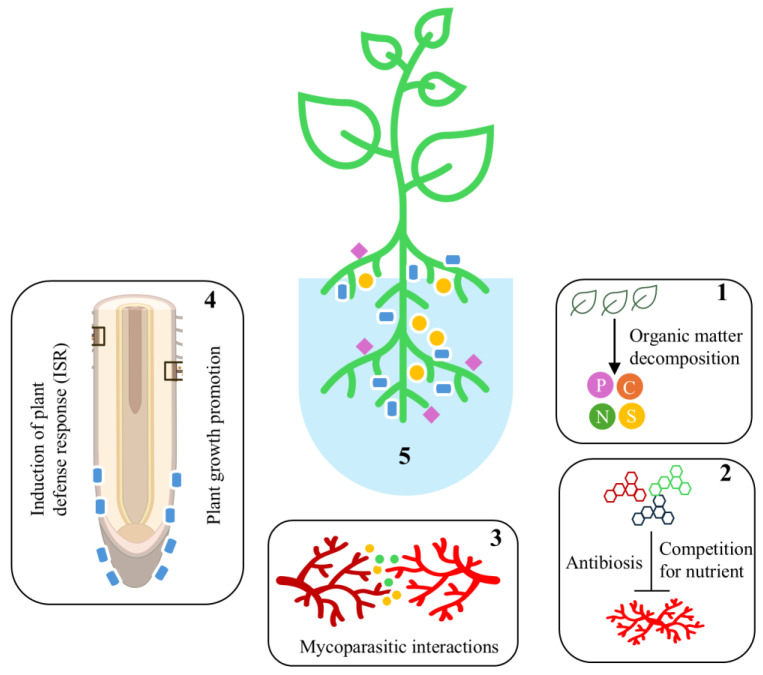
The primary mode of action of *Chaetomium globosum* as a plant growth promoter and biocontrol agent. (**1**) *Chaetomium globosum* is a key decomposer of soil organic matter that enhances soil fertility by increasing organic carbon, phosphorus, potassium, nitrogen, and pH levels in the rhizosphere. (**2**) Antibiosis through interference competition by producing secondary metabolites and enzymes. (**3**) Direct parasitism of fungal prey through secretion of hydrolytic enzymes, toxins, and other secondary metabolites. (**4**) Induction of systemic defense response and plant growth promotion by manipulating the host plant’s hormone biosynthesis. (**5**) *Chaetomium globosum* promotes beneficial microbes and their functioning in the rhizosphere and thereby enhances plant health.

Biocontrol of plant diseases is a complex process that involves the interaction between BCAs and pests or pathogens, plant hosts, and the environment. Hence, biocontrol relies on a range of mechanisms, including direct parasitism of pathogens (hyperparasitism), exploitation competition for nutrients and space, and interference competition through antibiosis [6,47]. For this, BCAs regulate their genetic machinery and produce a variety of secondary metabolites, hydrolytic enzymes, and small secreted proteins [48,49]. Small RNA [sRNAs]-mediated RNA silencing has been shown to regulate the production of such compounds and enzymes [50,51,52,53,54]. In addition, BCA can establish itself in plant tissues and live endophytically, triggering induced systemic resistance [47]. Based on the available literature, it is evident that interference competition through antibiosis is the primary mechanism employed by *C. globosum* for biocontrol interactions. Here, we explore the contribution of these mechanisms to the biocontrol ability of *C. globosum* (Figure 1).

### 3.1. Antibiosis

The production of antifungal metabolites and enzymes is considered an essential factor in interference and exploitative competition as well as hyperparasitism [47,49]. *Chaetomium globosum* is known for producing a variety of secondary metabolites with diverse biological activities [3]. However, this review focuses on the secondary metabolites identified for their antifungal activity against plant pathogenic fungi and nematodes.

Antibiosis is regarded as a crucial biocontrol trait of *C. globosum* and has attracted significant attention. Evidence of the antagonistic ability of *C. globosum* has mainly been found through in vitro assays, including dual-culture and culture filtrate tests using crude extracts and purified metabolites. For example, an in vitro dual-culture test revealed a strong antagonistic effect of *C. globosum* against the foliar pathogens of several plant species, including rice, maize, wheat and tomato [11,34,55,56,57,58]. Additionally, *C. globosum* is an effective antagonists against soil-borne pathogens causing root rot and wilt, including *Phytophthora* spp. [10,59,60] and *Fusarium* spp. [61,62].

Several metabolites have been isolated and identified from various *C. globosum* strains [1,3,7]. Chaetoglobosins and Chaetoviridins are the most studied metabolites for their antifungal activities against plant pathogens. Chaetoglobosins are one of the major secondary metabolites produced by *C. globosum*. These metabolites are known for several biological activities, including antifungal and nematocidal [1,63,64]. The biosynthetic gene cluster (BGC) for Chaetoglobosins has been identified in *C. globosum*, which contains a polyketide PKS-NRPS gene (*cheA*), an enoyl reductase gene [*cheB*], two genes coding cytochrome P450 oxygenase (*cheD* and *cheG*), a FAD-dependent monooxygenase gene (*cheE*), and two genes encoding the putative Zn[II]~2~Cys~6~ transcription factors *(cheC* and chef) [65,66]. CgLaeA and CgcheR transcription factors positively regulate the biosynthesis of Chaetoglobosin. At the same time, the basic helix–loop–helix family regulator CgXpp1 and the putative C2H2 transcription factor CgTF6 have been identified as negative regulators [67,68,69,70]. Several studies have revealed that chaetoglobosins exhibit significant inhibitory activity against plant pathogenic fungi.

Chaetoviridins are another major compound isolated from *C. globosum* [39,71,72]. Chaetoviridins A and B have been shown for antifungal effect against *S. sclerotiorum, R. solani, Magnaporthe grisea*, *P. ultimum*, *Puccinia recondita* and *V. dahliae* [39,66,72,73,74]. Additionally, these compounds have been reported for their antifungal activity against *Botrytis cinerea*, *Phytophthora capsici*, *Fusarium graminearum*, and *Fusarium moniliforme* [39,72]. Another study found that *Chaetoviridin A* treatment applied to *V. dahlia* caused cell necrosis, mycelial deformation, production of reactive oxygen species and nitrous oxide, and inhibition of microsclerotia germination [39]. Chaetoviridin is a polyketide, and the BGC of Chaetoviridin biosynthesis has been identified and characterized, consisting of 16 genes including two core genes encoding the HR-PKS (*cazF*) and NR-PKS (*cazM*) [75], which contribute to the polyketide backbone biosynthesis.

Other metabolites such as 4-methyl-[1,5-dimethyl-4-hexenyl]-benzene, tetradecane, dodecane, hexadecane, β-bisabolene, and dimethyl-propyl-disulphide have been reported to have antifungal properties. These metabolites were found to be effective against soil-borne fungal pathogens *S. sclerotiorum, S. rolfsii, M. phaseolina,* and *F. oxysporum* using the dual-culture technique [76]. Similarly, ethyl acetate and methanol extracts of *C. globosum* containing twenty-six secondary metabolites showed strong antifungal activity against *S. sclerotiorum* [77].

Secondary metabolites—including chaetoglobosin A, chaetoglobosin B, chaetocin, chaetoviridin, and chaetomugilin produced by *C. globosum*—have also been shown to have nematocidal activity against the RKNs *M. incognita* and *Meloidogyne javanica* [64,78,79,80]. The culture filtrate of *C. globosum,* containing chaetoglobosin A and chaetomin showed a significant biocontrol effect against the RKN *M. incognita* in tomato [64]. Additionally, polysaccharides with antibacterial activities have also been identified in *C. globosum* [81,82].

### 3.2. Mycoparasitism and Hyperparasitism

Mycoparasitism is a lifestyle in which one fungus establishes parasitic interactions with another fungus. A fungus that can parasitize other fungi is called a mycoparasite, and the fungus that acts as a parasitized host is called a fungal host (mycohosts). If the host is also a parasite, e.g., a plant pathogen, the interaction is known as hyperparasitic interaction or hyperparasitism. This interaction can be biotrophic when a mycoparasite gains nutrients from the living host fungus or necrotrophic when a mycoparasite kills the host fungus and feeds on the dead fungal biomass [47].

There are limited reports on mycoparasitic interactions between *C. globosum* and fungal hosts. In these reports, the mycoparasitic behaviour of *C. globosum* is characterized by attachment to the hyphae of fungal hosts, coiling around them, and overgrowing on mycelial colonies of fungal hosts during agar plate interaction studies [10]. The hyphae of *C. globosum* have been shown to penetrate the hyphae of *Phytophthora nicotianae*, resulting in the degradation and discolouration of pathogenic colonies [10]. Hyperparasitism of wheat spot blot pathogen *Cochliobolus sativus* by *C. globosum* has also been reported during dual culture interactions [7]. Similarly, Moya et al. [83] demonstrated that *C. globosum* effectively parasitizes the mycelium of *B. sorokiniana,* the causative agent of spot blotch in barley, significantly reducing its viability and pathogenicity [83,84].

### 3.3. Competition for Nutrients and Space

*C. globosum* employs competitive exclusion as a strategy to establish itself in a particular environment, including the rhizosphere. It competes with phytopathogenic fungi for essential nutrients and space. This competition can inhibit the growth and development of pathogens by depriving them of the resources necessary for survival. In vitro studies have shown that *C. globosum* can rapidly colonize substrates, effectively outcompeting pathogens such as the potato late blight pathogen *P. infestans* [85]. By occupying the ecological niche first and utilizing available nutrients, *C. globosum* prevents the establishment and proliferation of the pathogen [83].

### 3.4. Chaetomium globosum Genomics and Transcriptomics

Currently, the public domain lacks comprehensive genomic information on *C. globosum,* with only a draft genome sequence available for the strain CBS 148.51, which has an estimated genome size of 34.3 MB and a predicted 11,124 protein-coding genes [86]. The absence of this crucial information represents a significant gap in our understanding of the mode of action of *C. globosum;* hampers accurate species identification and classification; and impedes the efficient utilization of *C. globosum* strains. Furthermore, the lack of genomic information is a barrier to understanding the biocontrol mechanism and identifying genetic markers associated with biocontrol traits of *C. globosum*. The potential benefits of this data are immense, as it could significantly enhance research, application, and knowledge-based improvement of *C. globosum* as a biocontrol agent.

Comparing global gene expression patterns, in addition to genomics, in *C. globosum* during interactions with fungal and plant hosts (compared to non-interaction control) can provide vital clues for better understanding the underlying mechanisms of biocontrol interactions. Transcriptome analysis of *C. globosum* during in vitro dual-culture interaction with the wheat spot blotch pathogen *B. sorokiniana* using RNA-seq showed upregulation of a high proportion of genes associated with secondary metabolite biosynthesis [84]. These include genes coding for polyketide synthase, non-ribosomal peptide synthase, terpene cyclase, squalene epoxidase, ubiquinone biosynthesis methyltransferase, and glycerol-3-phosphate dehydrogenase. This supports the consideration that interference competition through antibiosis is a vital mechanism of *C. globosum* biocontrol activity [4]. This result also underscores that the production of secondary metabolites is one of the primary mechanisms of the interference competition of *C. globosum*.

The gene associated with secondary metabolite production is often arranged in clusters and co-expressed [87]. Membrane transporters such as MFS [major facilitator superfamily] or ABC (ATP-binding cassette) membrane transporters are integral to secondary metabolite BGCs and contribute to the efflux of secondary metabolites after their biosynthesis [87]. A higher expression of secondary metabolite genes during antagonistic interactions is often accompanied by a higher expression of membrane transporters [49]. Transcriptome analysis by Darshan et al. [84] revealed higher expression of secondary metabolite biosynthesis genes coupled with increased expression of membrane transporters in *C. globosum* [84], indicating their potential role in secondary metabolite efflux. A higher expression of membrane transporters during antagonistic interactions is plausibly also related to the detoxification mechanisms of *C. globosum* against external toxic metabolites that might come from the host fungus as a defence response [49,88,89].

Cell wall-degrading enzymes, including chitinases, proteases, and glucanases, play an essential role in mycoparasitism [49]. Genes coding for hydrolytic enzymes such as glycosyl hydrolase [GH2, GH13, GH31 and GH81 family], chitinases, cellulases, b-1, 3-glucanases, glucan endo-1,3-beta-glucanase, and cellulase-binding domain-containing proteins and proteases (including peptidases and aminopeptidases) were significantly upregulated in *C. globosum* during interaction with *B. sorokiniana* [84]. A higher expression of these genes suggests their potential role in *B. sorokiniana* mycoparasitism. In summary, the findings of Darshan et al. [84] indicated the role of hydrolytic enzymes, secondary metabolites, and membrane transporters in the antagonistic interaction of *C. globosum* [84]. This supports the consideration that antibiosis and mycoparasitism are essential mechanisms of *C. globosum*’s biocontrol activity [4].

### 3.5. Induction of Plant Defense Response

The induction of the plant defense response by plant beneficial fungi is effective against various pathogens and pests and is called induced systemic resistance (ISR) [90,91]. ISR is often mediated by jasmonic acid (JA) and ethylene (ET) and is salicylic acid (SA)-independent [90]. However, emerging evidence suggests that systemic resistance triggered by certain strains of *Trichoderma*, such as *T. longibrachiatum,* also involves SA signalling pathways [92]. Root colonization by *C. globosum* triggers a defense response [93,94,95]. Preliminary evidence suggests that *C. globosum* may induce plant resistance as a biocontrol mechanism in wheat against the tan spot pathogen *Pyrenophora tritici-repentis* and spot blotch pathogen *B. sorokiniana* [58,96]. More definitive proof comes from the work of Singh et al. [94], which demonstrated that applying *C. globosum* to tomato roots significantly reduced early blight disease caused by the foliar pathogen *Alternaria solani* [94].

Transcriptomic analyses of host plants such as tomatoes, cotton and cucumber during interactions with *C. globosum* have shed light on the molecular mechanisms by which *C. globosum* induces disease resistance in plants [93,94,95]. Transcriptome analysis of the tripartite interactions between *C. globosum*, the root pathogen *V. dahliae*, and cotton plants revealed the induced expression of genes linked to flavonoid and phenylpropanoid biosynthesis pathways, MAPK [mitogen-activated protein kinase] signalling, glutathione metabolism, and plant–pathogen interactions [93]. The role of phenylpropanoids and flavonoids, along with MAPK signalling, is well established in the defense mechanisms of cotton [97,98,99]. Additionally, genes associated with hormone biosynthesis, such JA, ET, and SA, were induced in cotton plants treated with *C. globosum*. In contrast, abscisic acid, auxin, and gibberellin levels were significantly elevated in *C. globosum*-treated plants following inoculation with *V. dahliae* [93]. These findings indicate that *C. globosum* can induce a defence response in cotton plants through various mechanisms involving secondary metabolites and hormone signalling pathways.

Similarly, transcriptome analysis of tomato leaves and cucumber roots treated with *C. globosum* revealed an upregulation of genes associated with various pathways, including phenylpropanoid biosynthesis, plant hormone signal transduction, plant–pathogen interactions, and the MAPK signalling pathway [94,95]. Singh et al. [94] showed that genes involved in JA biosynthesis were upregulated, whereas those related to ET biosynthesis were downregulated. This suggests that JA-mediated signalling actively contributes to ISR in tomatoes colonized by *C. globosum*. Additionally, genes related to SA biosynthesis, such as phenylalanine ammonia-lyase (PAL) and phospholipase D (PLD), along with pathogenesis-related (PR) protein-coding genes were also upregulated in plants treated with *C. globosum* compared to untreated plants. These findings indicate that *C. globosum* can trigger both JA- and SA-dependent signalling pathways in tomato plants [94]. These findings were corroborated by the higher content of plant hormones, including indole-3-acetic acid, gibberellin, SA, and JA, in seedlings colonized by *C. globosum* compared to non-inoculated seedlings [95].

## 4. *Chaetomium globosum* as a Plant Growth Promoter

Emerging evidence has shown that *C. globosum* can colonize plant roots and live as endophytes [14,95,100]. Root colonization by *C. globosum* promotes plant growth and development and the overall yield of a variety of crops [14,95,100]. For example, *C. globosum* application promotes plant length and biomass of *Salvia miltiorrhiza* [100]; enhances plant growth promotion; and also enhances plant biomass and yield of tomato, cotton, cucumber, and Chinese crab and apple plants [94,95,101,102]. Similarly, the application of *C. globosum* to potato tuber and direct soil application resulted in significantly higher seed germination and enhanced plant length and tuber yield in the presence of *R. solani* under field and greenhouse conditions [103]. The plant growth promotion effect of ethyl acetate and methanolic extracts of *C. globosum* on *Brassica* seedlings has also been reported [77].

The plant health promotion effect of *C. globosum* is attributed to the regulation and homeostasis of plant hormone and secondary metabolite biosynthesis, such as phenylpropanoid and chicoric acid [93,94,95,104]. This is supported by transcriptome analysis whereupon root colonization by *C. globosum* induced the expression of genes associated with the biosynthesis of phytohormones, photosynthesis, and secondary metabolite biosynthesis and metabolisms [93,94,95]. *Chaetomium globosum* is reported to produce phosphatases and phytases, mobilize phosphorus, and enhance the production of castor, wheat, and pearl millet [101].

## 5. Effect of *C. globosum* Application on Soil Health and Plant Microbiome

Beneficial microbes enhance soil health, as indicated by increased soil enzymatic activity, enhanced organic carbon transformation, and increased microbial diversity. Key soil health indicators include soil sucrase, catalase, urease, and acid phosphatase, as well as total nitrogen, accessible potassium, phosphorus, pH, and total potassium [105]. In addition to improving nutrient availability and acquisition and modulating growth and defence-related phytohormones, biocontrol fungi promote plant health by influencing the rhizosphere microbiome. This includes suppressing plant pathogens through various mechanisms and enhancing the relative abundance and diversity of indigenous soil growth-promoting microbial taxa. Such changes may be driven by microbial resource competition, antagonism due to antibiosis, interactions with plants that result ISR in plants [106]. For example, the application of biocontrol *Trichoderma guizhouense* to the rhizosphere of cabbage plant and *T. harzianum* to maize rhizosphere resulted in disease suppression and plant health promotion, which is attributed to the enrichment of soil-borne beneficial microbes [107,108].

Soil application of *C. globosum* resulted in a significant increase in soil fertility indices such as enzyme activities, phosphorus, potassium, and nitrogen content and pH in the cotton rhizosphere compared to the non-application control [108]. Similarly, *C. globosum* application increased soil enzyme activity and soil actinomycete bacteria [102]. Microbial community sequencing revealed that the abundance of soil-borne plant pathogenic fungi, including cotton wilt pathogens *Fusarium* spp. and *V. dahliae*, decreased in the cotton rhizosphere after *C. globosum* application. At the same time, the abundance of plant-beneficial bacteria, including *Sphingomonas, Bacillus, Pseudomonas* and *Rhizobium,* was increased. This shift in microbial community composition is plausibly attributed to the higher antibiosis activity of *C. globosum* towards fungi, consequently reducing competition for nutrients and space between fungi and bacteria and resulting in higher bacterial abundance [108]. Reports from Ma et al. suggest that soil application of *C. globosum* promotes soil health and microbiome functioning, which is one of the primary biocontrol mechanisms. More profound knowledge of the plant rhizosphere microbiome augmented with *C. globosum* will provide a better understanding of the interactions of *C. globosum* with the microbial community in a natural environment. This knowledge can offer unexplored opportunities to enhance biocontrol efficacy under field conditions and help to develop innovative biocontrol methods, such as selecting compatible microbial agents to develop consortia of BCAs.

## 6. Taxonomy of *Chaetomium globosum*

Gustav Kunze first described the genus *Chaetomium* in 1817, with *C. globosum* identified as the first species [109]. The fungus belongs to Phylum Ascomycota, Order Sordariales, Family *Chaetomiaceae*. Since first identification, many *Chaetomium* species have been identified based on morphological characteristics. The morphological traits used for classifying *Chaetomium* species include features of sexual structures, such as ascomata hairs, ascomata walls, and the morphology and arrangement of asci and ascospores. These include globose, ovate or obovate ostiolate ascomata covered with distinctive hairs; erect, flexuous, or coiled ascomatal hairs; an ascomatal wall with intricate texture; evanescent, clavate, or slightly fusiform asci; and limoniform or bilaterally flattened ascospores with an apical germ pore [110,111]. Ascospores’ characteristics include brown to grey-brown pigment, with one to two germ pores occasionally appearing as a black, dark, and tacky mass [110,111]. Based on these characteristics, the genus *Chaetomium* consists of over 400 proposed species epithets and approximately 270 accepted species [112]. While morphological characteristics have aided in the identification of several new species within this genus, many researchers consider the taxonomic classification based on these traits to be inconsistent and unreliable [70].

The absence of genomic information significantly hampers our understanding of this fungus’s genetic diversity and classification. Addressing this gap is essential for advancing our research and conservation efforts. However, studies in the last few decades that focus on multigene phylogenetic analysis combined with morphological traits have made significant progress in the species delimitation of *C. globosum* [70,110,111]. The loci used in these studies include ITS (internal transcribed spacers), LSU (D1/D2 domains of the 28S nrDNA), *rpb1* and *rpb2* (RNA polymerase II first and second largest subunit genes), *tef1* (elongation factor 1-α), and *tub2* (β-tubulin gene). The combination of morphological analysis and multigene phylogenetic studies has resulted in the identification of 43 species within the genus *Chaetomium* [70]. Among the genetic markers used, *tub2* and *rpb2* were the most effective at distinguishing between species in the *Chaetomium* complex [70,111]. To assess the genetic diversity of *C. globosum,* Darshan et al. [113] utilized RNA sequencing data and developed 22 expressed sequence tag–simple sequence repeat (EST-SSR) markers. These markers were used to analyze the genetic diversity among 15 *C. globosum* strains isolated from various ecological niches, including wheat leaves, grains, dung, and Dolichos seeds [113]. Additionally, other molecular markers such as Universal Rice Primer PCR (URP-PCR), Random Amplified Polymorphic DNA (RAPD), Amplified Fragment Length Polymorphisms (AFLPs), and Inter-Simple Sequence Repeat [ISSR] markers have also been employed to evaluate the diversity of *C. globosum* [8].

## 7. Distribution of *Chaetomium globosum*

*C. globosum* is a widely distributed fungus in diverse environments from deserts to Arctic permafrost and is one of the predominant fungal taxa in soil fungal communities worldwide [114]. It thrives in nutrient-rich soil, organic compost, and decaying materials, acting as a saprotroph that breaks down complex organic matter [80]. Known for its strong cellulolytic activity, *C. globosum* decomposes cellulose effectively and is commonly found in decaying wood, leaf litter, and composting sites. Its decomposition promotes nutrient cycling, enhancing soil health and fertility [115,116]. In addition to its presence in natural environments, *C. globosum* has been discovered indoors. It flourishes under high-humidity conditions and can colonize various substrates, including wallpaper, textiles, and wooden structures, often leading to biodeterioration and structural damage. Moreover, this fungus poses a risk to food products, where it can cause spoilage and reduce quality [104]. Interestingly, strains of *C. globosum* have also been isolated from living animal hosts and the tissues of various plants, including gymnosperms, dicots, and monocots, indicating its endophytic lifestyle. This ability to inhabit diverse biological systems emphasizes its role as an ecological generalist. The adaptability of *C. globosum* to various ecological niches is likely attributed to its capacity to produce a wide range of secondary metabolites and enzymes, further enhancing its survival and ecological success.

## 8. *Chaetomium globosum* Cultivation and Formulation

In vitro culturing of microorganisms is vital in investigating their biology, mode of action, and environmental interactions. The knowledge generated from such studies helps to formulate strategies for their potential applications, including plant disease management. *Chaetomium globosum* exhibits various degrees of growth and sporulation on culture media commonly used in microbiological research under laboratory conditions. These media include potato dextrose agar, oatmeal agar, cornmeal agar, malt extract agar, Czapek-Dox agar, sabouraud agar, and Richard medium. *C. globosum* can grow on these media at 20–40 °C with an optimum growth rate of 25 °C to 35 °C [117,118,119,120]. Ease of in vitro culturing offers an excellent opportunity to understand *C. globosum* biology under various laboratory conditions where various growth parameters such as temperature, pH, and nutrient availability can be precisely managed for optimal functioning. For example, optimized production of cellulolytic enzymes and secondary metabolites and knowledge-based improvement of fungal traits contribute to biocontrol application and formulation for large-scale application under greenhouse and field conditions. In addition, adjusting growth conditions [e.g., different carbon and nitrogen sources, light conditions, temperature and pH] can lead to the identification of novel compounds that might not be produced under standard conditions. In addition, in vitro systems can facilitate genetic studies, including mutagenesis and genetic engineering, which can help to understand the underlying mechanisms of *C. globosum* as a biocontrol agent.

The formulation process involves several critical steps: selecting an appropriate type of formulation, mass production of the fungal spores, stabilization for prolonged shelf life, and choosing effective application methods [121]. *Chaetomium globosum* can be formulated for large-scale application under glasshouse and field conditions, leveraging its biocontrol properties against various plant pathogens. *C. globosum* can be prepared in different formulations, such as liquid, powder, and granular forms [122]. Liquid formulations of *C. globosum* involve suspending or emulsifying the fungal spores in a liquid carrier. These formulations are particularly advantageous for their ease of application and ability to provide uniform coverage on plant surfaces [123]. Additionally, the liquid medium can help to maintain the viability of the spores during storage and application [124]. Powder formulations consist of *C. globosum* spores mixed with inert carriers such as talc, clay, or other mineral powders. These carriers provide a stable environment for the spores, facilitating their storage and handling [125]. Powder formulations can be dusted directly onto plants or soil or reconstituted with water for spray applications. The stability and ease of storage of powder formulations make them suitable for immediate and long-term use [126].

Granular formulations incorporate *C. globosum* spores into granular carriers, which can be applied directly to the soil. This formulation is particularly effective for targeting soil-borne pathogens, as the granules can deliver the spores directly to the root zone of plants [127]. Granular formulations are applied using standard granular applicators and can be designed to release the spores gradually, ensuring prolonged protection against soil-borne diseases [121]. The talc-based formulation of *C. globosum* effectively inhibited various soil-borne pathogens, including *Fusarium* spp. and *P. infestans* [12,30,128]. Formulations based on *C. globosum* proved more effective than chemical treatments in managing soil-borne potato diseases [103,129].

## 9. Prospects

**Figure 2:**
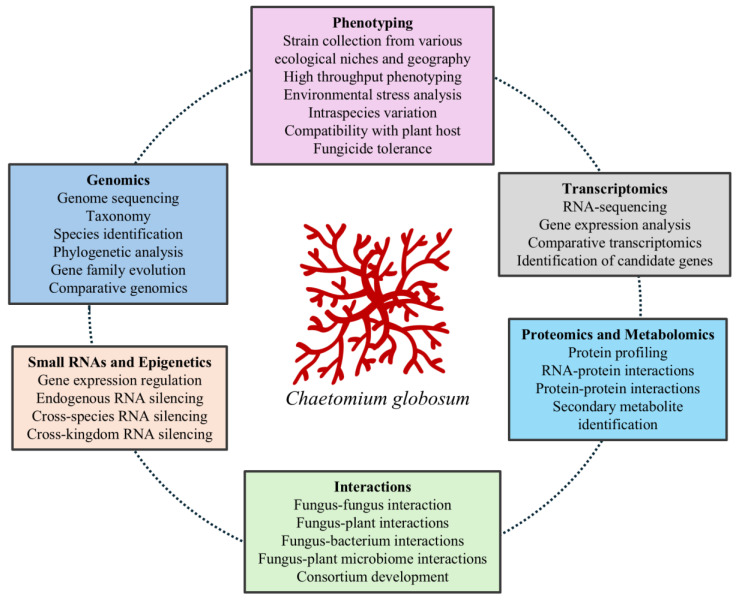
Summary highlighting the existing knowledge gaps and the integration of omics technology to explore the biocontrol mechanisms of *C. globosum* for a well-informed approach to enhancing its biocontrol efficacy.

High efficacy and consistency are crucial for the successful integration of BCAs into crop production systems. One of the bottlenecks is the reduced efficacy of BCAs in the field compared to their higher efficacy in laboratory and controlled small-scale experiments. This is attributed to lack of deeper insights into the ecology and biology of BCAs, along with their interactions with natural microbes and plant hosts under field conditions. Recent advances in omics and molecular biology technology and microbiome studies have enabled researchers to overcome these challenges. Despite advances in Omics technologies, however, knowledge about *C. globosum* genomics and the underlying mechanisms of its interaction with the environment and fungal and plant hosts remains elusive. Addressing this knowledge gap can significantly contribute to better integration of *C. globosum* as a BCA in crop production systems. For instance, genomic information on *C. globosum* can aid in its identification, clarify its taxonomic position, and elucidate its relationships with other BCAs. Whole-genome sequencing can also facilitate the exploration of gene copy numbers and gene family evolution and help to identify key genes associated with biocontrol traits. This includes genes responsible for secondary metabolite production, host–microbe interactions, and signalling pathways that mediate growth, development, reproduction, and ecological niche adaptation. Furthermore, genomic data can provide opportunities to investigate genetic similarities and differences between *C. globosum* and other fungal BCAs, particularly in terms of their life strategies and modes of action as BCAs. Additionally, the availability of whole-genome sequenced data from a larger number of *C. globosum* strains can facilitate the development of DNA-based markers for strain identification [Figure 2].

Fungal BCAs exhibit significant intraspecific variations concerning their traits related to nematode antagonisms [130] and their compatibility with different host plants [131,132]. Thus, selecting a strain with greater biocontrol potential and compatibility with fungal and plant hosts is crucial to ensure effective biocontrol outcomes. To identify *C. globosum* strains with optimum biocontrol compatibility and consistent efficacy, a comprehensive screening process involving diverse *C. globosum* strains from various ecological environments can prove invaluable. By assessing their efficacy against a broad spectrum of plant pathogens while matching them with specific host plants, the most effective strains for biocontrol of a particular pathogen or enhancing the health of certain crops can be identified. Understanding the compatibility of BCAs, such as *C. globosum,* with plant pathogens and host plants presents an exciting opportunity to incorporate these findings into advanced plant breeding programs for optimized biocontrol potential. This integration would enable the strategic selection of plants that display desirable traits such as high yield and superior quality and exhibit enhanced disease resistance due to their compatibility with effective biocontrol agents. 

Emerging evidence on the role of sRNAs-mediated RNA silencing in fungal interactions relevant to biocontrol opens new avenues for biocontrol research [50,51,52,53,54]. This includes the role of sRNAs as a master regulator of secondary metabolite production, which is a crucial trait of fungal BCAs. Since the antagonistic ability of *C. globosum* is mainly attributed to its ability to produce a range of secondary metabolites, identifying sRNA regulating secondary metabolite production in *C. globosum* may be useful for optimizing its secondary metabolite production and therefore its antagonistic potential. The genetic and genomic information of *C. globosum* coupled with knowledge of RNA silencing can be leveraged for its genetic improvement using genetic transformation systems, including CRISPR-Cas9 [Clustered Regularly Interspaced Short Palindromic Repeats–CRISPR-Associated Protein 9] genome-editing technology.

## Figures and Tables

**Table 1:** A list of plant diseases reported to be managed by *Chaetomium globosum* in greenhouse and field conditions.

Disease	Plant	Pathogen	References
Anthracnose	Black pepper	*Phytophthora palmivora,*	Soytong et al., 2021 [22]
Anthracnose	Mango	*Colletotrichum gloeosporioides*	Noiaium, 1999 [23]
Ascochyta blight	Chickpea	*Ascochyta rabiei*	Rajkumar et al., 2005 [9]
Apple scab	Apple	*Venturia inaequalis*	Andrews et al., 1983 Cullen and Andrews, 1984 [17,18]
Sooty blotch	Apple	*Phyllachora pomigena*	Davis et al., 1992 [19]
Spot blotch	Barley	*Bipolaris sorokiniana*	Aggarwal et al., 2004 [7]
Spot blotch	Wheat	*Bipolaris sorokiniana*	Aggarwal et al., 2004 [7]
Basal rot	Corn	*Sclerotium rolfsii*	Soytong, 2010 [24]
Citrus leaf miner	Tangerine	*Phytophthora parasitica*	Soytong et al., 2021 [22]
Coffee Wilt	Coffee	*Fusarium roseum*	Van and Soytong, 2015 [25]
Damping off	Sugarbeet	*Pythiumultimum*	Walther and Gindrat, 1988 [26]
Damping off	Cotton	*Pythium ultimum*	Di Pietro et al., 1992 [27]
Damping off	Peas	*Pythium*	Khali et al., 2020 [28]
Damping off	Radishes	*Rhizoctonia solani*	Aggarwal et al., 2004 [7]
Wilt	Tomato	*Fusarium oxysporum*	Madbouly et al., 2017 [29]
Grape white rot	Grape	*Coniothyriumdiplodiella*	Zhang et al., 2013 [1]
Grey blight	Coffee	*Pestalotia* spp.	Phong et al., 2014 [30]
Late blight	Potato	*Phytophthora Infestans*	Shanthiyaa et al., 2013 [12]
Leaf anthracnose	Coffee	*Colletotrichum gloeosporioides*	Vilavong and Soytong, 2017 [31]
Leaf spot	Rice	*Curvularialunata*	Tathan, 2012 [32]
Leather rot	Strawberry	*Phytophthora cactorum*	Mouden et al., 2016 [33]
Peach rot	Peach	*Rhizopus stolonifer*	Zhang et al., 2013 [1]
Rice blast	Rice	*Pyricularia oryzae*	Kasem and Quimio, 1989 [34]
Root rot	Pomelo	*Phytophthora palmivora*	Hung et al., 2015 [10]
Seed rot	Radish	*Alternaria raphani*	Vannucci and Harman, 1987 [20]
Seed-corn maggot	Squash	*Fusarium solani*	Hubbard et al., 1982 [35]
Seedling blight	Oat	*Fusarium* spp.	Tveit and Wood, 1955 [36]
Seedling mortality	Chili	*Sclerotium rolfsii* *Colletotrichum capsici*	Sultana et al., 2012 [37]
Stem canker	Soybean	*Diaporthephaseolorum* var. *meridionalis*	Dhingra and Santana, 2003 [21]
Spot blotch	Wheat	*Drechslerasorokiniana*	Aggarwal et al., 2004 [7]
Tea wilt	Tea	*Fusarium roseum*	La et al., 2016 [38]
Wilt	Cotton	*Verticillium dahliae*	Zhang et al., 2021 [39]
White rot	Onion	*Sclerotium cepivorum,*	Ali, 2020 [40]
Root rot	Citrus	*Phytophthora nicotianae* *Pythium ultimum*	Hung et al., 2015 Kean et al., 2010 [10,41]
Leaf spot	Tomato	*Alternaria alternata*	Fayyadh and Yousif, 2019 [11]
Root rot	Date palm	*Rhizoctonia solani,* *Fusarium oxysporum,* *Fusarium chlamydosporum*	Lewaa and Zakaria, 2023 [13]
Root rot	Corn	*Fusarium roseum*	Kommedahl and Chang, 1968. Kommedahl and Mew, 1975 [15,16]

## Data Availability

No new data were created or analyzed in this study. Data sharing is not applicable to this article.
